# Risky sexual practice and associated factors among antiretroviral therapy attendees in public health facilities, Wolaita Zone, South Ethiopia: a multi-center cross-sectional study

**DOI:** 10.3389/frph.2024.1470574

**Published:** 2024-11-19

**Authors:** Sisay Petros Salato, Befekadu Bekele Besha, Esayas Aydiko Amele, Temesgen Lera Abiso

**Affiliations:** ^1^ Damot Gale District Health Office, Boditi, Ethiopia; ^2^ School of Public Health, College of Health Science and Medicine, Wolaita Sodo University, Wolaita Sodo, Ethiopia; ^3^ School of Nursing, College of Health Science and Medicine, Wolaita Sodo University, Wolaita Sodo, Ethiopia

**Keywords:** risky sexual practice, HIV, adults, ART, Ethiopia

## Abstract

**Background:**

The majority of people living with Human Immunodeficiency Virus (HIV) are in low- and middle-income countries, particularly in sub-Saharan Africa. Increased risky sexual practice puts people living with the human immune virus at higher risk of acquiring sexually transmitted infections other than the human immune virus and unplanned pregnancies. Sexually transmitted infections, particularly viral hepatitis (B and C), significantly impair antiretroviral therapy and the clinical outcome of the co-infected individual, leading to increased morbidity and mortality. The purpose of this study was to investigate the prevalence of risky sexual practices among antiretroviral therapy (ART) attendees in public health facilities within the Wolaita Zone of South Ethiopia.

**Methods:**

From September to October 2023, a facility-based cross-sectional study was conducted among adult people living with HIV on ART in the Wolaita zone. Data was collected through a pretested and structured questionnaire. Six diploma nurses were trained to collect data. Systematic sampling techniques were used to select a total of 398 ART patients. Data were collected by Open Data Kit (ODK) and analyzed with SPSS Version 25. Binary and multiple logistic regression analyses were used. All the variables with a *P*-value of 0.25 associated with risky sexual practices are considered candidate variables. Multicollinearity was checked. The fitness of the model was tested by the Hosmer-Lemshow goodness of fit test. Finally, statistical significance was declared at a *p*-value of 0.05.

**Result:**

A total of 398 respondents took part in this study. The prevalence of risky sexual practices in the past six months was 174 (43.7%) at 95% of the CI (38.9–48.7). The result of multiple logistic regression analysis showed that not disclosing HIV status (AOR = 1.8, 95% CI: 1.1–3.31), alcohol drinking (AOR = 3.1, 95% CI: 1.66–0.023), and poor social support (AOR = 1.9, 95% CI: 1.75–3.9) were statistically significantly associated with risky sexual practices.

**Conclusion:**

This study revealed that the risky sexual practice among ART clients was high and disclosure status, social support, and alcohol use were factors associated with risky sexual practice. The governmental and non-governmental bodies have to strengthen social support for ART clients, disclosure status for ART clients, and counseling to avoid alcohol.

## Introduction

Risky sexual practices are defined as inconsistent and/or no condom use with a partner who is HIV-negative or has an unknown sero-status (1). According to 2023 UNAIDS report, 39 million people were living with HIV globally and sixty-five percent of people living with HIV (PLHIV) reside in sub-Saharan Africa (2). Ethiopia is one of the sub-Saharan countries that is worst affected by the HIV/AIDS pandemic. In 2019, the national HIV prevalence was estimated to be 0.9% (3). Increased risky sexual behavior puts PLHIV at higher risk of acquiring sexually transmitted infections (STIs) other than HIV and unplanned pregnancies; it has been reported that STIs, especially viral hepatitis (B and C), have a significant negative impact on ART and the clinical outcome of the co-infected person, including increased morbidity and mortality (4).

Ethiopia is primarily focusing on people uninfected with HIV and the sexual risk practices of HIV-infected individuals who did not receive due attention (5).

HIV/AIDS remains a major public health problem. According to UNAIDS global statistics, about 37.7 million [30.2 million–45.1 million] people worldwide were living with HIV, and 1.5 million people became newly infected with HIV in 2020. About 27.5 million people were taking antiretroviral therapy (ART), and an estimated 680,000 people died from AIDS-related illnesses in 2020 (1).

Globally, unprotected sex among HIV-positive people on ART became one of the problems that aggravated HIV transmission. The worldwide focus of HIV prevention efforts was largely on people who were uninfected with HIV/AIDS, but the sexual behavior of HIV-infected persons did not receive any serious attention for a variety of reasons (2). Evidence shows that 20%–80% of people living with HIV/AIDS continue to engage in risky sexual practices in sub-Saharan countries (3). Study conducted in Addis Ababa, Ethiopia showed that risky sexual practices that may further transmit the virus put them at risk of contracting secondary sexually transmitted infections and lead to problems with drug resistance (4).

Initially, a diagnosis of HIV infection counted as a death sentence. In that context, the sexual life of those infected persons seemed a secondary issue, which made prevention focused on sexual behavior hard to imagine. Furthermore, the conviction that stigmatization should be avoided also precluded an interest in the sexual behavior of HIV-infected persons (1). Although many HIV-infected individuals avoid risky sexual practices, substantial numbers of them continue to engage in HIV-transmission-risk behaviours, which puts them at risk of reinfection by HIV strains resistant to ARV drugs or acquiring other sexually transmitted diseases (STDs), which hasten AIDS progression (2).

The majority of people newly infected with HIV in sub-Saharan Africa are infected during unprotected heterosexual intercourse (including paid sex) and onward transmission of HIV to newly born and breastfed babies. Having unprotected sex with multiple partners remains the greatest risk factor for HIV acquisition in this region (3).

Risky sexual practice among people receiving ART is an area of concern; hence, it is the major effective driver of the HIV epidemic. Among people living with HIV (PLHIV), these behaviors are common and potentially expose their partners to a risk of disease; for HIV-positive partners, these habits expose them to a real risk of infection by other strains of IV (4). Risky sexual practice among people receiving ART is an area of concern; hence, it is the major effective driver of the HIV epidemic. Among people living with HIV (PLHIV), these behaviors are common and potentially expose their partners to a risk of disease; for HIV-positive partners, these habits expose them to a real risk of infection by other strains of HIV. The magnitude of unprotected sexual practice among PLHIV is high in sub-Saharan Africa; more than 1 in 3 PLHIV were engaged in risky sexual practice (5).

Early sexual debut, multiple sexual partnerships, limited and inconsistent condom use, sex under the influence of alcohol, childhood marriages, and transactional cross-generational sex are the main risky behaviors currently driving the HIV epidemic in Ethiopia (6). Risky sexual practices increase the risk of HIV/AIDS, unintended pregnancy, unsafe abortion, and psychosocial problems (7). Therefore, the purpose of this study was to investigate the prevalence of risky sexual practices among antiretroviral therapy (ART) attendees in public health facilities within the Wolaita Zone of South Ethiopia.

## Methods and materials

### Study design and setting

An institution-based cross-sectional study was conducted from September to October 2023 in Wolaita zone, South Ethiopia, at a distance of 328 km from the capital city of the country, Addis Ababa, and 151 km from the regional capital city of Hawassa. There are about 67 health centers, six primary hospitals, and one comprehensive hospital. And there are 5,502 health workers in the Wolaita zone. There are about 18 ART sites. The total number of PLHIV currently on ART in the zone is 4,174.

#### Source population

The source populations were all adult ART attendees in public health facilities in Wolaita Zone.

#### Study population

Systematically selected ART attendees who were found in randomly selected public health facilities in the Wolaita zone.

#### Inclusion criteria

ART attendees in the age group ≥18 years who have been practicing sexual activities in the past six months were included in the study.

#### Exclusion criteria

Those who are seriously ill and unable to communicate verbally were excluded from the study**.**

#### Sample size determination

The sample size for the study was calculated by using single population proportion formula considering 95% level of confidence, 5% margin of error and 10% non-response rate, the proportion of practice is 40.9% (1). For finite population we use correction formula (*N* < 10,000) $\;\frac{n}{1+\frac{n}{N}}=372/(1+372/4174)=342$ after adding non-response of 10%, the final sample size calculated was 403.

### Sampling technique

First, 30% of health facilities were selected from all health facilities that provide ART service to ensure representativeness of all health facilities after stratification by hospital and health center. Accordingly, six health facilities (2 hospitals from 7 to 4 health centers from 11) are randomly selected. Then, the calculated sample size (*n* = 403) was proportionally allocated to selected health facilities. We used systematic random sampling method to select the study participants until the allocated number of participants in each selected health facility is reached or obtained. The sampling interval was determined based on the number of patients who came for follow-up to the ART clinic each month. The average number of patients who came to the ART clinic for follow-up during the study period was estimated at 1,187. By considering the monthly client flow for follow-up, the sampling interval was two.

### Data collection procedures and quality assurance

A structured questionnaire was developed to collect socio-demographic data, relationship-related factors, social-related factors, and behavioral factors. The client chart was reviewed to get data about medically related factors. Trained six diploma nurses collected the data. The questionnaire was prepared in English and translated into the Wolaitgna language. To check whether the translation was consistent with the language, the questionnaire was back translated to English by another language expert. A pre-test was done in 5% of ART patients in the study area, which would not be included in the actual study, to assess the content and approach of the questionnaire, and necessary adjustments were made before actual data collection. The questionnaire was also tested for internal consistency (reliability) by Cronbach's alpha test. The reliability coefficients for attitude and knowledge items had Cronbach's alpha of 0.841 and 0.701, respectively. The completeness, consistency, and accuracy of the collected data were examined by the principal investigator on a daily basis.

Training of supervisors and data collectors was given for one day before data collection. They were given information on ethical issues such as the need to observe confidentiality and obtain informed consent from participants prior to administering study tools. The overall supervision of data collection process was done by the principal investigator. Meetings were held to address problems and clarify issues that hampered the collection of good data with assistants who were found to have problems.

#### Data entry & analysis

The data was collected by ODK and exported to SPSS 25 statistical software for analysis. After cleaning the data in SPSS for inconsistencies and missing values, descriptive statistics were done. All the variables with *P*-values of 0.25 and risky sexual practices in the bivariable analysis were candidates for the final multivariable logistic regression model. Multi-Collinearity was checked using the Variance Inflation Factor (VIF); values 10 were included in the model. The fitness of the model was tested by the Hosmer-Lemshow goodness of fit test (*p*-value of 0.05). In the multivariable analysis, a value of *P* 0.05 was considered statistically significant. An adjusted odds ratio (AOR) with a 95% confidence level would be used to show the strength of association between dependent and independent variables. Finally, the result was presented through narration, tables, and figures.

### Study variables

#### Dependent Variable

• Risky sexual practice.

#### Independent variables

Socio-demographic characteristics, relationship factors, behavioral factors, medical related factors knowledge and attitude on HIV transmission, prevention, condom use and others social support factors.

### Operational definitions

#### Risky sexual practice

Having one or more of the following practices during the past six months prior to the date of data collection: having multiple sexual partners, casual sex, sex with out or inconsistent use of a condom even with a regular partner, sex with the influence of a substance like alcohol, khat (5).

#### Casual sex

Defined as sexual behavior that occurs between people who are not romantically involved and suggests a lack of emotional attachment, commitment, or familiarity. Examples include having sex while casually dating, having one-night stands, prostitution, swinging, and having friendships with benefits or sporadic sexual behavior that takes place outside of a committed partnership (8, 9).

#### Knowledge

To assess the level of knowledge, respondents who score greater than or equal to the mean are considered to have good knowledge, and those who score less than the mean are considered to have poor knowledge.

#### Attitude

To assess the level of attitude, respondents who score greater than or equal to the mean are considered to have a good attitude, and those who score less than the mean are considered to have a poor attitude.

#### Social support factors

Social support was measured using the OSLO 3-item social support scale. The sum score ranges from 3 to 14, with high values representing a strong level and low values representing a poor level of social support. The oslo-3 sum score can be broken down into three types of social support: 3–8 poor social support, 9–11 moderate social and 12–14 strong social support.

## Result

The study was conducted on 398 participants, including adult ART attendants from 4 selected public health centers and 2 hospitals, with a 99% response rate. The results mainly fall into two categories: the status of risky sexual practice and associated factors.

### Socio demographic characteristics

The majority of ART attendees, 309 (77.6%), were female. The age interval of 30 to 39 years accounts for 188 (47.2%) of the entire ART population, and the mean age of respondents was 35 years with a standard deviation of 8 (358). The marital status of respondents was: 248 (62.3%) were married. Educational status respondents were 133 (33.4%) and 89 (22.4%), both of whom were able to read and write and attended primary school. Housewives made up 110 (27.6%) of ART attendants. Regarding the income of ART patients, the majority of respondents (222, or 55.8%) reported getting ≥3,000 Birr per month. The residences of 343 (86.2%) ART attendants were urban. One hundred ninety-nine (49.7%) and 154 (38.7%) were protestant and orthodox followers, respectively (Table 1).

**Table 1:** Socio-demographic characteristics of ART patients Wolaita zone Ethiopia, 2023.

Variable		Frequency	Percent
Sex	Male	89	22.4
	Female	309	77.6
Age	18–29	78	19.6
	30–39	188	47.2
	≥40	121	30.4
Marital status	Single	66	16.6
	Married	248	62.3
	Divorce	35	8.8
	Widowed	49	12.3
Educational status	unable read and write	44	11.1
	able read and write	133	33.3
	Primary (1–8)	89	22.4
	Secondary (9–12)	88	22.1
	college and above	44	11.1
Occupation	Governmental	89	22.4
	house wife	110	27.6
	Merchant	77	19.3
	Private	78	19.6
	Farmer	33	8.3
	Others	11	2.8
Monthly income	<1,500 ($26)	132	33.2
	1,500–2,999 ($26–$52)	44	11.1
	≥3,000 ($52)	222	55.7
Residence	Urban	343	86.2
	Rural	55	13.8
Religious	Protestant	198	49.7
	Orthodox	154	38.7
	Muslim	33	8.3
	Catholic	13	3.3

### Assessment of medical related factors

Majority respondents among ART attendants: 266 (66.8%) were ≥48 months in ART follow-up clinic. The CD4 count was 376 (94.5%), which was greater than 200 cells/m^3^. In terms of condom use, 365 (91.7%) of respondents used condoms. Thirty-three (8.3%) used condoms before being confirmed HIV positive, and they had safe sex discussions with health workers. One hundred forty-three (34.9%) of the respondents disclosed their results to the community. Three hundred seventy-six (94.5%) respondents got counseling on the importance of protecting from new strain and received the risk reduction strategy. Of the total respondents, 55 (13.8%) also had an HIV prevention discussion or support group.

### Assessment of attitude of ART patients

**Figure 1:**
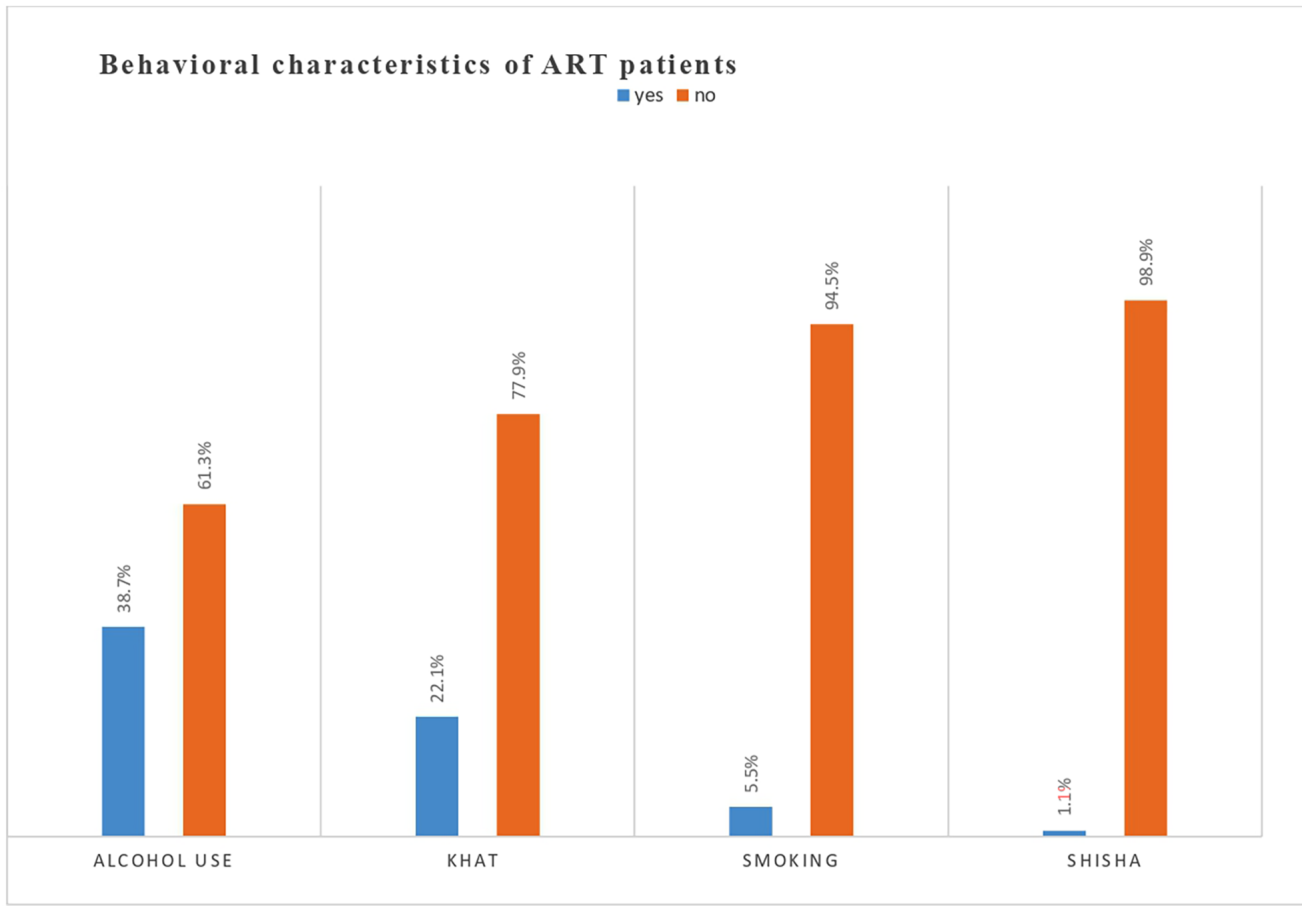
Behavioral characteristics of ART patients in Wolaita Zone, South Ethiopia.

The result of the attitude assessment showed that more than half the respondents, 210 (52.8%), had a positive attitude for preventive aspects of risky sexual practices and 188 (47.2%), had a negative attitude for preventive aspects of risky sexual practices (Figure 1).

### Behavioral factors

**Figure 2:**
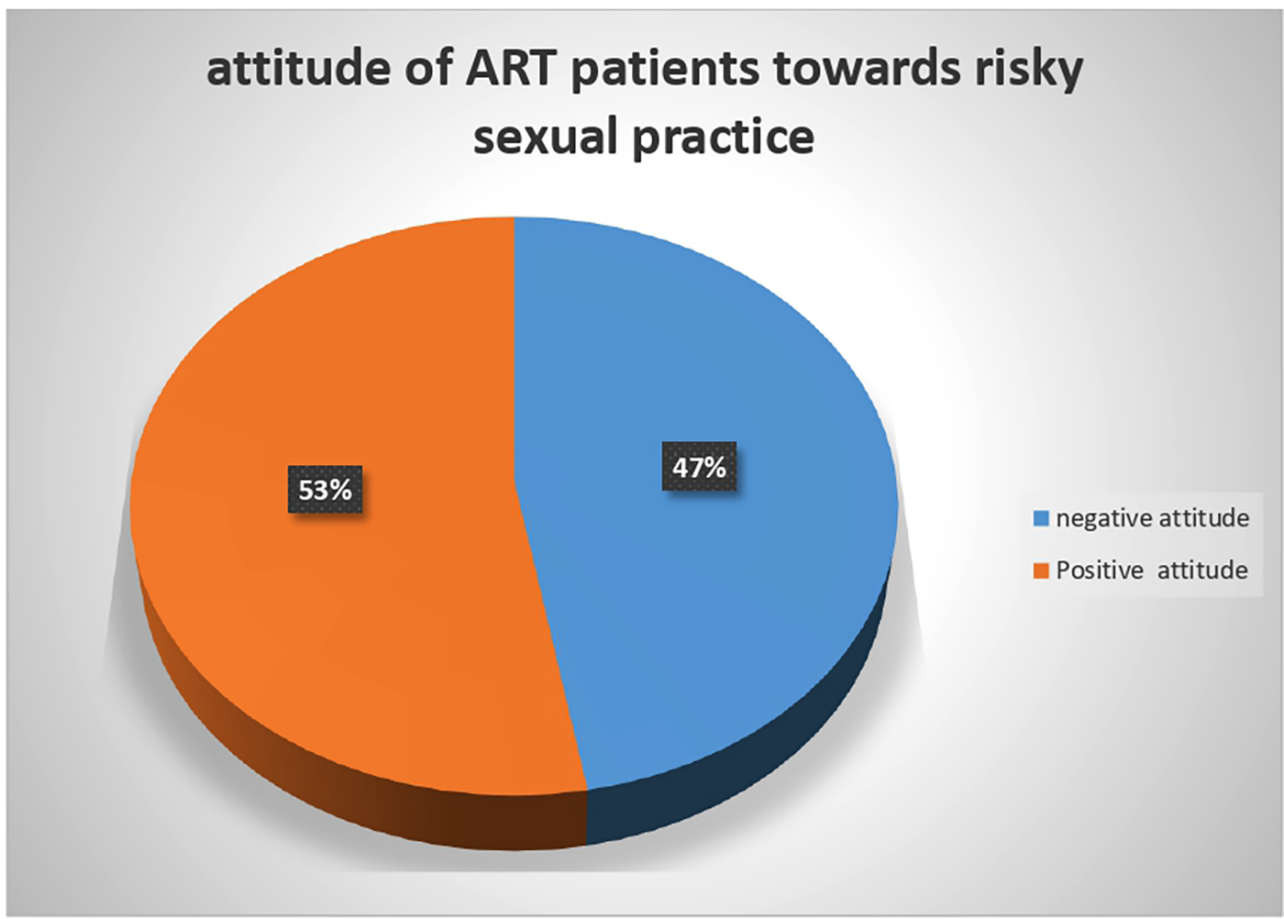
Attitude of ART attendees towards risky sexual practice in Wolaita Zone, South Ethiopia.

Of the 244 responders, the majority (61.3%) did not consume alcohol. In terms of khat use, 88 (22.1%) of those surveyed did so. Twenty-two people (5.5%) and four (1.1%) smokers of shisha were present (Figure 2**).**

### Knowledge of ART patients

We assessed the knowledge of risky sexual practices of the participants by using seven items, five of the items were yes or no answer items and the rest two were items with choices with two and there options. According to the result of the assessment, 276 (69.3%) of respondents had good knowledge, while 122 (30.7%) of them had poor knowledge (Table 2).

**Table 2:** Knowledge on risky sexual practice of ART patients Wolaita zone Ethiopia, 2023.

S.no	Variable		Frequency	Percent
1	Do you the transmission method of HIV/AIDS	Yes	331	83.2
		No	67	16.8
2	Do you know the prevention of HIV/Aids	Yes	387	97.2
		No	11	2.8
3	If yes which method of prevention	Use condom	331	83.2
		Absence	67	16.8
4	Do you know the importance of condom	Yes	398	100.0
		No	11	2.8
5	If yes what is the importance of condom	To prevent HIV Aids	332	83.4
		To prevent unwanted pregnancy	55	13.8
		I don't know	11	2.8
6	Do you know the benefit of ART	Yes	376	94.5
		No	22	5.5
7	Do you know the importance of viral load	Yes	343	86.2
		No	55	13.8
	Level of knowledge	Good knowledge	276	69.3
		Poor knowledge	122	30.7

### Risky sexual practice among ART attendants

This study revealed that from the total of 398 ART participants, 174 (43.7%) ART patients practiced risky sexual practice and 224 (56.3%) didn't practice risky sexual practices. From the total of 398 ART patients, 159 (40%) had sex without a condom, 33 (8.3%) had sex under the influence of alcohol which was relatively less frequent compared with other risky sexual practice, 132 (33.2%) ART patients have multiple sexual partner, 110 (27.6%) had causal sex and 99 (24.9%) used condom consistently with partner.

## Partner related characteristics

According to the result of the assessment, 244 (61.3%) had sexual partners in the past 6 months, and 211 (53%) had one partner. Regarding the type of sexual partner, 167 (42%) were regular sexual partners. One hundred sixty-six (41.7%) knew the HIV status of their sexual partner, and 111 (45.9%) had an unknown HIV status. One hundred sixty-seven (68.4%) disclosed their status to their sexual partners.

## Social support factors

According to the result of the assessment, 209 (52.5% of respondents) had poor social support, while 123 (30.9%) of them had strong social support (Table 3).

**Table 3:** Social support status of ART client's Wolaita zone, 2023.

Variable		Frequency	Percent
How many people are so close to you that you can count on them if you have a serious problem?	None	143	35.9
	1–2	99	24.9
	3–5	101	25.4
	>5	55	13.4
How much concern do people show for what you are doing?	A lot of concern and interest	132	33.2
	Some concern and interest	22	5.5
	Uncertain	22	5.5
	Little concern and interest	99	24.9
	No concern and interest	123	30.9
How easy is it to get practical help from neighbors if you should need it?	Very easy	176	44.2
	Easy	22	5.5
	Possible	33	8.3
	Difficult	101	25.4
	Very difficult	66	16.6
Level of social support	Poor social support	209	52.5
	Moderate social support	66	16.6
	Strong social support	123	30.9

### Factors associated with risky sexual practice

Results of bivariate logistic regression analysis showed that marital status, age of respondents, residence, having sexual partners, disclosing HIV status to the community, getting counselling, the attitude of ART attendants towards risky sexual practices, using alcohol, knowledge, and social support were candidate variables (which had a *p*-value of less than 0.25). In the multivariable logistic regression analysis, disclosure status, social support, and alcohol use were statistically significant.

In this study, participants who reported not disclosing their HIV status were 1.8 times more likely to risky sexual practice compared to those who disclosed their status. AOR = 1.8, 95% CI (1.3–7.1). Respondents addicted to alcohol were 3.1 times more likely to engage in risky sexual practices as compared to non-alcoholics. AOR = 3.1, 95% CI (1.64–6.023). In this study, participants who had poor social support were 1.9 times more likely to engage in risky sexual practices when compared to those with strong social support. AOR = 1.9, 95% CI (1.1–3.31) (Table 4).

**Table 4:** Associated factors for risky sexual practice among ART clients Wolaita zone, 2023.

Variable		Risky sexual practice		OR (95% confidence interval)		*p*-value
		Yes (*n* = 174)	No (*n* = 224)	COR (95% CI)	AOR (95% CI)	
Sex	Male	44 (49.4%)	45 (50.6%)	1.4 (0.84–2.2)	3.1 (0.3–7.4)	0.97
	Female	130 (42.1%)	179 (57.9%)	1	1	
Age of respondents	18–29	38 (44.7%)	47 (55.3%)	1.3 (0.75–2.3)	1.5 (0.54–4.13)	0.435
	30–39	92 (46.7%)	105 (53.3%)	1.4 (0.9–2.3)	0.953 (0.52–1.76)	0.878
	≥40	44 (37.9%)	72 (62.1%)	1	1	
Marital status	Single	48 (72.7%)	18 (27.3%)	16 (6.1–42.1)	23.02 (0.71–75.23)	0.167
	Married	100 (40.3%)	148 (59.7%)	4.05 (1.8–9.4)	7.97 (0.55–24.9)	0.154
	Divorce	19 (54.3%)	16 (45.7%)	7.13 (2.5–20.2	8.1 (0.4–27.7)	0.43
	Widowed	7 (14.3%)	42 (85.7%)	1	1	
Residence	Rural	158 (46.1%)	185 (53.9%)	2.1 (1.12–3.87)	1.6 (0.7–3.6)	0.194
	Urban	16 (29.1%))	39 (70.9%)	1	1	
Had sexual partners	Yes	115 (47.1%)	129 (52.9%)	1.4 (0.95–2.17)	1.9 (0.98–3.65)	0.56
	No	59 (38.3%)	95 (61.7%)	1	1	
Disclose HIV status to community/people	Yes	101 (39.6%)	154 (60.4%)	1	1	
	No	73 (51.0%)	70 (49.0%)	1.6 (1.05–2.4)	**1.8** (1.3–7.1)*	**0**.**032**
Get counseling	No	169 (44.9%)	207 (55.1%)	2.8 (1.01–7.68)	1.9 (0.4–8.8)	0.426
	Yes	5 (22.7%)	17 (77.3%)	1	1	
Alcohol use	No	79 (32.4%)	165 (67.6%)	1	1	
	Yes	95 (61.7%)	59 (38.3%)	3.4 (2.2–5.13)	**3.1** (1.64–6.023)*	**0**.**002**
Attitude	Positive attitude	109 (58.0%)	79 (42.0%)	3.1 (2.04–4.7)	1.2 (0.11–0.45)	0.32
	Negative attitude	65 (31.0%)	145 (69.0%)	1	**1**	
Knowledge	Good	117 (42.4%)	159 (57.6%)	1		
	Poor	57 (46.7%)	65 (53.3%)	1.2 (0.78–1.83)	1.849 (0.483–5.6)	0.435
Social support	Poor	117 (42.4%)	93 (44.5%)	3.01 (1.9–4.9)	**1.9** (1.1–3.31)*	**0**.**029**
	Moderate	22 (33.3%)	44 (66.7%)	1.21(0.64–2.3)	0.7(0.34–1.67)	0.426
	Strong	36(29.3%)	87(70.7%)	1	1	

Bold values denote AOR and *p*-value for significantly associated factors.

* Significant association.

## Discussion

This study revealed that risky sexual practices among the ART patients was 43.7% and disclosure status, social support, and alcohol use were factors associated with risk of sexual practice.

One hundred seventy-four (43.7%) of ART patients had a risky sexual practice. This finding was relatively similar to the study finding at Gondar University Referral Hospital 3 8% (6), in Ronda 38% (7), Kembata tembaro 40.9% (1) and Addis Abeba public hospital (39.1%) (10). On the other hand, this finding is lower than the findings of other studies in Alibena (61.9% male and 86.3% female) (11), western Oromia 56.9% (12), in Ethiopia (79.8%) (13) and of study participant involved in unprotected sexual practice. And this finding is higher than the findings of other studies in Jamaica (12%) (14) and Vietnam, 3.6% and 5.6% of patients had sexual intercourse with casual partners and sex workers, respectively (15), Ethiopia, (30.4%) (4), in Debrezeit Town 15.8% (2), Northwest of Ethiopia 22.2% (16) and in Nekemet Referral hospital (32.9%) (3). Such differences in the risk of sexual practice being higher or lower than this study could be due to the time of the study period, socio-demographic factors, like educational, economic, and cultural factors of the current study area and study settings, composite scoring, and the type of healthcare facilities.

In this study, factors associated with risky sexual practice among ART attendants—disclosure status, social support, and alcohol use—were statistically significant.

In this study, participants who reported not disclosing their HIV status were 1.8 times more likely to practice risky sexual practices compared to those who disclosed their HIV status [AOR = 1.8, 95% CI (1.3–7.1)]. The possible explanation for this finding could be the fact that disclosure of ART clients could decrease risky sexual practices due to the fact that ART patients are more likely to disclose their status.

Another variable, respondents who were addicted to alcohol were 3.1 times more likely to engage in risky sexual behavior than non-alcoholics OR = 3.1, 95% CI (1.64–6.023).This finding is similar to the study conducted in Northern India, which showed that high-risk sexual behavior was nine times more common among patients addicted to alcohol as compared to non-alcoholics (17), in Addis Ababa, respondents who had a history of substance abuse were involved in risky sexual practices more than those who had a history of substance abuse (2), north-west Ethiopia showed that frequent use of alcohol was indecently predictive of unprotected sexual practice (4), and the study conducted in public hospital of Ethiopia revealed that, alcohol consumption was highly associated with unprotected sexual practice (13). The possible explanation for this finding could be the fact that alcohol consumption increases the risky sexual practices of ART patients. Due to the intoxication of ART patients, risky sexual practices were more likely to be engaged, or the fact that alcohol use can hinder the thinking and decision-making abilities about safe sex.

In this study, participants with poor social support were 1.9 times more likely to engage in risky sexual practices when compared to those with strong social support (AOR = 1.9, 95% CI (1.1–3.31).

This study, similar to the study conducted in A Systematic Review of Global Literature on Social Support and HIV Related Risk Practices, suggested that social support was positively associated with consistent condom use (safe sex) among PWHIV (18), in the U.S. indicated that the link between positive support networks and the avoidance of high-risky sexual practices in HIV-positive individuals (19), PWHIV with a high level of perceived HIV-specific support were more likely to consistently use condoms during sexual intercourse (19), A cross-sectional study was done on the effect of social support in the lives of adults with HIV/AIDS (20) and other cross-sectional and prospective predictors study done on unsafe sex among HIV positive individuals in Clark County show that social support has a positive effect on maintaining safe sex (20).

The possible explanation for this finding could be the fact that strong social support decreases the risky sexual practices of ART patients. This may involve identifying an appropriate support or peer group, providing educational materials and counseling, or connecting them to other families or individuals for emotional support.

## Limitation of the study

This is a cross-sectional study, and the design does not determine the direction of causality. The sensitive nature of sexuality may result in social desirability bias, which may underestimate the prevalence of risky sexual practices. Again the study could not address the imbalance of male to female ratio which needs further investigation.

## Conclusion

In conclusion, the results of the study revealed that the risk of sexual practice among ART clients was high. Disclosure status, social support, and use of alcohol were factors associated with risky sexual practice. Therefore strengthening the disclosure status of ART patients by giving health education via health workers, health program leaders and other governmental and non-governmental organizations working on HIV prevention and treatment, the follow-up of ART patients to decrease the risky sexual practice, the social support of ART clients in order to avoid emotional stress and counseling the ART client to avoid alcohol consumption are needed.

## Data Availability

The original contributions presented in the study are included in the article/Supplementary Material, further inquiries can be directed to the corresponding author.
